# Human skeletal muscle nitrate and nitrite in individuals with peripheral arterial disease: Effect of inorganic nitrate supplementation and exercise

**DOI:** 10.14814/phy2.15531

**Published:** 2022-12-02

**Authors:** Barbora Piknova, Mary N. Woessner, Joaquin Ortiz de Zevallos, William E. Kraus, Mitch D. VanBruggen, Alan N. Schechter, Jason D. Allen

**Affiliations:** ^1^ Molecular Medicine Branch, NIDDK National Institutes of Health Bethesda Maryland USA; ^2^ Institute for Health and Sport (IHES) Victoria University Melbourne Australia; ^3^ Department of Kinesiology, School of Health and Human Development University of Virginia Charlottesville Virginia USA; ^4^ Duke Molecular Physiology Institute, Duke University School of Medicine Durham North Carolina USA; ^5^ Division of Cardiovascular Medicine, School of Medicine University of Virginia Charlottesville Virginia USA

**Keywords:** inorganic nitrate, Nitrite, Skeletal Muscle, Exercise Capacity, Peripheral Arterial Disease

## Abstract

Skeletal muscle may act as a reservoir for N‐oxides following inorganic nitrate supplementation. This idea is most intriguing in individuals with peripheral artery disease (PAD) who are unable to endogenously upregulate nitric oxide. This study analyzed plasma and skeletal muscle nitrate and nitrite concentrations along with exercise performance, prior to and following 12‐weeks of exercise training combined with oral inorganic nitrate supplementation (EX+BR) or placebo (EX+PL) in participants with PAD. Non‐supplemented, at baseline, there were no differences in plasma and muscle nitrate. For nitrite, muscle concentration was higher than plasma (+0.10 nmol.g^−1^). After 12 ‐weeks, acute oral nitrate increased both plasma and muscle nitrate (455.04 and 121.14 nmol.g^−1^, *p* < 0.01), which were correlated (r = 0.63, *p* < 0.01), plasma nitrate increase was greater than in muscle (*p* < 0.01). Nitrite increased in the plasma (1.01 nmol.g^−1^, *p* < 0.05) but not in the muscle (0.22 nmol.g^−1^) (*p* < 0.05 between compartments). Peak walk time (PWT) increased in both groups (PL + 257.6 s;BR + 315.0 s). Six‐minute walk (6 MW) distance increased only in the (EX+BR) group (BR + 75.4 m). We report no substantial gradient of nitrate (or nitrite) from skeletal muscle to plasma, suggesting a lack of reservoir‐like function in participants with PAD. Oral nitrate supplementation produced increases in skeletal muscle nitrate, but not skeletal muscle nitrite. The related changes in nitrate concentration between plasma and muscle suggests a potential for inter‐compartmental nitrate “communication”. Skeletal muscle did not appear to play a role in within compartment nitrate reduction. Muscle nitrate and nitrite concentrations did not appear to contribute to exercise performance in patients with PAD.

## Graphical abstract

Nitric oxide (NO) affects a plethora of biological functions including blood flow, cellular respiration and has been proposed as an exercise ergogenic. Skeletal muscle may act as a reservoir for N‐oxides following inorganic nitrate supplementation. This idea is appealing in individuals with peripheral artery disease (PAD) who are unable to endogenously upregulate NO and have poor functional capacities. We report no substantial gradient of nitrate (or nitrite) from skeletal muscle to plasma, suggesting a lack of reservoir‐like function in participants with PAD. Oral nitrate supplementation produced increases in skeletal muscle nitrate, but not skeletal muscle nitrite. Changes in nitrate concentration between plasma and muscle suggests a potential for inter‐compartmental nitrate “communication”. Skeletal muscle did not appear to play a role in within compartment nitrate reduction. Muscle nitrate and nitrite concentrations did not appear to contribute to exercise performance in PAD.

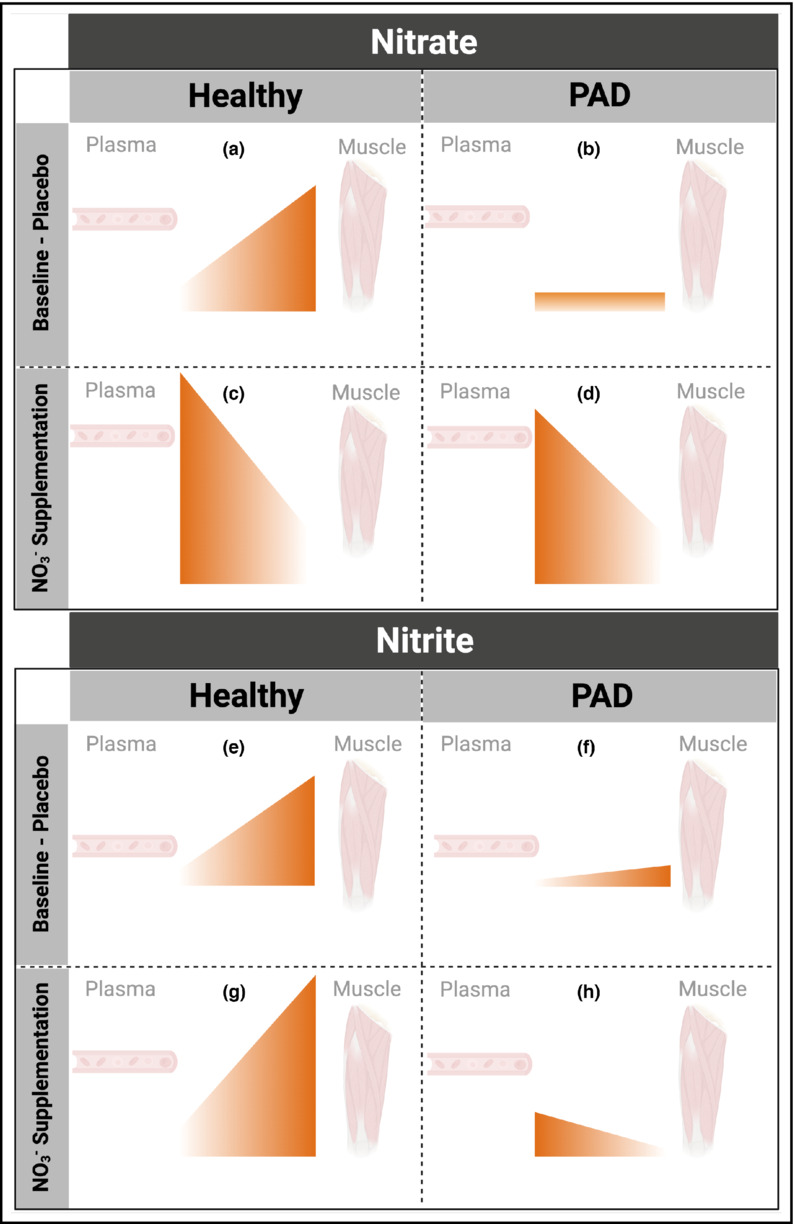

## 1 INTRODUCTION

Nitric oxide (NO) is a lipid soluble, diatomic gas that affects a plethora of biological functions from immune defense and neurotransmission to blood flow regulation and exercise performance. In cohorts with vascular pathologies, endothelial dysfunction limits the ability to endogenously upregulate NO production. Thus, approaches to exogenously increase NO bioavailability have focused on these populations to improve cardiovascular health and physical function. Oral inorganic nitrate supplementation is an accepted strategy to increase the circulating pool of bioavailable NO. Despite the now well studied entero‐salivary pathway of N‐oxides conversion and transport, our understanding of the potential role of skeletal muscle tissue as a reservoir for NO‐bioactivity is limited.

It is established that ingestion of oral inorganic nitrate can substantially increase plasma nitrate and nitrite concentrations, and that plasma nitrite can be subsequently reduced to NO, especially in conditions of low oxygen availability. Recent studies suggest that skeletal muscle tissue may also contribute to the production, storage, and metabolism of nitrate in mammals, however data is limited (Nyakayiru et al., 2017; Park, Thomas, Schechter, & Piknova, 2021; Piknova et al., 2015; Wylie et al., 2019). The premise for the role of the skeletal muscle as a reservoir is appealing, as under resting, non‐supplemented conditions in rodents there is a gradient of nitrate concentration from several skeletal muscle groups to the vascular compartment and to the liver (Park, Thomas, Schechter, & Piknova, 2021; Piknova et al., 2015). Moreover, the skeletal muscle contains relatively high concentrations of neuronal NO Synthase (nNOS, NOS1), which is indispensable for muscle integrity and contractile performance (Percival, 2011). Given that skeletal muscle comprises approximately 40%–50% of the human mass, even small concentrations of stored N‐oxides could potentially serve as a very large reservoir of nitrate and nitrite that can be preferentially released to augment circulating levels (Park, Thomas, Schechter, & Piknova, 2021).

Dietary inorganic nitrate supplementation leads to increases in nitrate concentrations across muscle and blood compartments in both rodents and humans (Gilliard et al., 2018; Kadach et al., 2022; Park, Thomas, Schechter, & Piknova, 2021; Piknova et al., 2015; Wylie et al., 2019). In rats a 5‐day high nitrate diet increased gluteus nitrate by 2.4‐fold, extensor digitorum longus (EDL) 4.3‐fold and soleus 3.3‐fold (Park, Thomas, Schechter, & Piknova, 2021). Similarly, in healthy young adults, an acute dose of inorganic nitrate increased vastus lateralis (VL) nitrate several‐fold (Kadach et al., 2022; Wylie et al., 2019). While these data are intriguing, the studies in humans are limited. Moreover, an internal reservoir of N‐oxides could potentially be most beneficial to individuals with cardiovascular disease characterized by endothelial dysfunction and an inability to upregulate NO bioavailability endogenously. These individuals often have low functional capacities which may be most amenable to potential ergogenic effects (Sim et al., 2021; Woessner, McIlvenna, et al., 2018). To date there are no studies outlining changes in skeletal and plasma nitrate and nitrite concentrations along with changes in exercise performance following oral nitrate supplementation in humans with documented vasculopathies.

Based on the above analysis, it is reasonable to hypothesize that some forms of cardiovascular diseases associated with endothelial dysfunction would also lead to dysfunctional nitric oxide metabolism. In the present study we examined the effect of acute (2.5 h) dietary nitrate supplementation on plasma and skeletal muscle nitrate concentrations and exercise performance in participants with Peripheral Arterial Disease (PAD) following 12‐weeks of exercise training combined with oral inorganic nitrate supplementation (EX+BR) or placebo (EX+PL). We also examined the association among nitrate and nitrite concentrations in both the plasma and skeletal muscle tissue, and their relationships to exercise performance in these individuals, to clarify potential intercompartmental signaling.

## 2 METHODS

**FIGURE 1:**
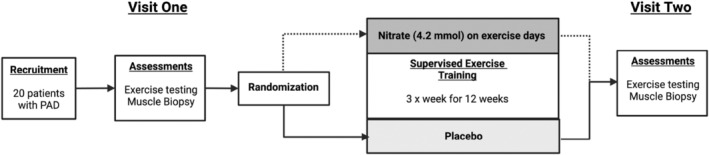
Study design schematic. Following the screening assessments (*testing visit 1‐conducted over two separate days*), subjects followed a 12‐weeks rehabilitation program in combination with either oral inorganic nitrate (EX+BR) or nitrate‐depleted (EX+PL) supplementation. At the end of the 12‐week period the completed the same battery of test done on visit 1.

The data presented in this manuscript is a sub‐study analysis from data previously collected from randomized trial involving participants with PAD. The trial examined the effect of inorganic nitrate supplementation in the form of beetroot juice (BR) versus placebo (PL) (James White Drinks) on exercise performance. The study was approved by the appropriate Human Research Ethics Committee and are registered on *ClinicalTrials.gov* (NCT01785524). The full protocol and the main outcomes have been published previously (Woessner et al., 2017; Woessner, VanBruggen, et al., 2018). The relevant methodology is detailed in brief below with simplified study designs relevant to this manuscript presented in Figure 1.

### 2.1 The peripheral arterial disease cohort

Twenty four participants aged 40–85 years with symptomatic PAD were enrolled in randomized controlled, double‐blind parallel designed trial (Woessner et al., 2017). The primary aim of the trial was to determine whether a 36‐session supervised exercise rehabilitation program in combination with consuming oral inorganic nitrate (EX+BR) 2.5 h prior to each session would produce superior benefits over exercise plus placebo (EX+PL) in pain free walking time (Figure 1 and testing protocol). The dosage of 4.2 mmol nitrate was taken from previous studies at the time that demonstrated average increases in plasma nitrite above 400 nM and most likely to provide an ergogenic benefit. The 2.5‐h timing of beverage administration prior to exercise training and testing is required to allow sufficient time for plasma nitrite concentrations to peak. The process requires the oral nitrate to be swallowed, absorbed into the blood. It is then sequestered into the salivary glands, secreted into the oral cavity where commensal bacteria convert the nitrate to nitrite and it is again swallowed and absorbed into the plasma (Kenjale et al., 2011). A subset of 20 participants from the study also completed two gastrocnemius muscle biopsies (baseline and 12 weeks) which are included in the data presented here (see Table 1 for participant inclusion and exclusion criteria and Table 2 for demographics and medications). It is noteworthy that the data points shown are from baseline testing, prior to randomization, at which no supplementation occurred and at the end of 12 weeks post randomization and with participants still supplemented with BR (4.2 mmol nitrate) or PL approximately 2.5 h prior to data/sample collections on the day of testing. Data presented from the parent trial (Woessner, VanBruggen, et al., 2018) was taken from a subsequent visit 1 week after suspension of supplementation. The exercise outcomes are not different between visits.

**TABLE 1:** Patient inclusion and exclusion criteria.

Inclusion criteria
Aged between 40 and 80 years.
2 Diagnosed peripheral artery disease (ankle‐brachial index <0.90) with intermittent claudication.
3 No major changes in medications for at least 3 months
Exclusion criteria
Foot ulcers, advanced neuropathy, gangrene, or other musculoskeletal condition that could limit exercise performance.
2 Type 1 diabetes or glycated hemoglobin >8.5%.
3 A major cardiovascular event within the previous 6 weeks or a planned hospitalization within the next 2 months.
4 Any cardiovascular condition that impacts safety of completing a cardiopulmonary exercise test, including history of significant left main or three‐vessel coronary artery disease (>70% stenosis), recent myocardial infarction (6 weeks), chest pain during cardiopulmonary exercise test, or >2 mm ST depression during exercise; foot ulcers/advanced neuropathy or other musculoskeletal condition that could limit exercise performance.
5 Allergy to beets or proton pump inhibitors.
6 Refusal or inability to abstain from the use of proton pump inhibitors for 24 h prior to testing.

**TABLE 2:** Participant demographics and medications.

Variable	Placebo (*n* = 11, ♂ = 5)	Nitrate (*n* = 8, ♂ = 7)
Age, mean ± SD, year	72 ± 8	68 ± 9
Weight ± SD, kg	80.8 ± 12.5	85.4 ± 16
Height ± SD, cm	170.4 ± 7.3	174.5 ± 8.1
Diabetic, *n* (%)	2 (18%)	5 (63%)
Medications		
ASA/Plavix (blood thinner)	11 (100%)	8 (100%)
B‐blocker	7 (64%)	7 (86%)
Ca^2+^ channel blocker	3 (27%)	6 (75%)
Ace‐Inhibitor	8 (73%)	5 (63%)
HMG‐CoA reductase inhibitors	10 (91%)	6 (75%)
Glucose controlling Meds	2 (18%)	5 (63%)
Metformin	1 (9%)	5 (63%)
Proton pump inhibitor	3 (27%)	1 (13%)

### 2.2 Plasma nitrate and nitrite analysis

Five milliliter blood samples were drawn from the antecubital vein into a nitrate free syringe and separated into five 1 ml Eppendorf tubes containing 5 μL 1 to 1000 heparin. These tubes were then centrifuged for 3 min at 5000 rpm. The resultant plasma was then pipetted into separate 1 ml Eppendorf tubes, and immediately snap frozen in liquid nitrogen and stored at −80°C until analysis. Plasma nitrate and nitrite concentrations were measured by gas‐phase chemiluminescence using a NOA 280i Sievers nitric oxide analyzer, as previously described per manufacturer's instructions (Sievers Instruments) (Kenjale et al., 2011; Pinder et al., 2008).

### 2.3 Skeletal muscle nitrate and nitrite analysis

Skeletal Muscle samples were collected from the gastrocnemius muscle using a Bergstrom needle with suction (on a separate day to the maximal treadmill exercise test) as previously reported (Woessner et al., 2017). All muscle samples were kept frozen until analysis of muscle nitrate and nitrite (Park, Thomas, Wylie, et al., 2021). Muscle samples were initially weighed (20–150 mg wet weight), mixed with nitrite preserving solution (K_3_Fe(CN)_6_, N‐ethylmaleimide, water, Nonidet P‐40), as described in (Park, Thomas, Wylie, et al., 2021) and homogenized using a GentleMacs homogenizer (Miltenyi Biotec Inc). Proteins were then precipitated using cold methanol. Specifically, thawed samples were mixed with ice‐cold methanol (dilution 1:3 sample: methanol) and then centrifuged at 11,000 g for 5 min at 4°C. Supernatants were collected and immediately used to determine nitrate and nitrite content by chemiluminescence. The nitrite and nitrate content were determined by a Sievers gas‐phase chemiluminescence NO analyzer (Sievers 280i Nitric Oxide Analyzer, GE Analytical Instruments). Nitrate and nitrite data are presented as nanomoles per gram wet weight of tissue.

#### 2.3.1 Exercise training and testing protocols

Following baseline testing, participants were randomly assigned to consume either 70 ml (4.2 mmol NO_3_^−^) beetroot (EX+BR) or an identical nitrate‐depleted placebo (EX+PL), 2.5 hours before each exercise training visit (3× per week for 12 weeks), as previously detailed (Woessner, VanBruggen, et al., 2018). Each training session included at least 30 min of actual walking, with the intensity tailored to each participant's initial baseline maximal graded exercise test results. When claudication pain became moderately severe (3–4/5 on a 5‐point claudication pain scale), they would step off the treadmill and rest until the pain subsided (rest periods were not included in the 30‐min exercise time). Typically, after a patient was able to walk 8 to 10 min at the initial workload, the grade was increased by 0.5%, or the speed increased by 0.1 mph as tolerated.

Each exercise testing visits included, on separate days a 6 min walk distance (6MWD) test and maximal treadmill exercise test with a 12‐lead ECG to measure peak walk time (PWT) and claudication onset time (COT) (Woessner et al., 2017).

### 2.4 Statistics

Data were analyzed using independent samples and repeated measures t‐tests, respectively. Relationships among plasma nitrite and nitrate, muscle nitrate and nitrite, and exercise performance were assessed using Pearson's product moment correlation coefficients. All statistical analysis was conducted using GraphPad Prism Version 9.3 (GraphPad Software, www.graphpad.com). GraphPad Prism was also utilized for the creation of all graphs and figures. Data from both studies are reported as mean ± SD unless otherwise stated, with *p* < 0.05 required for statistical significance.

## 3 RESULTS

### 3.1 Baseline plasma and muscle nitrate and nitrite concentrations

**FIGURE 2:**
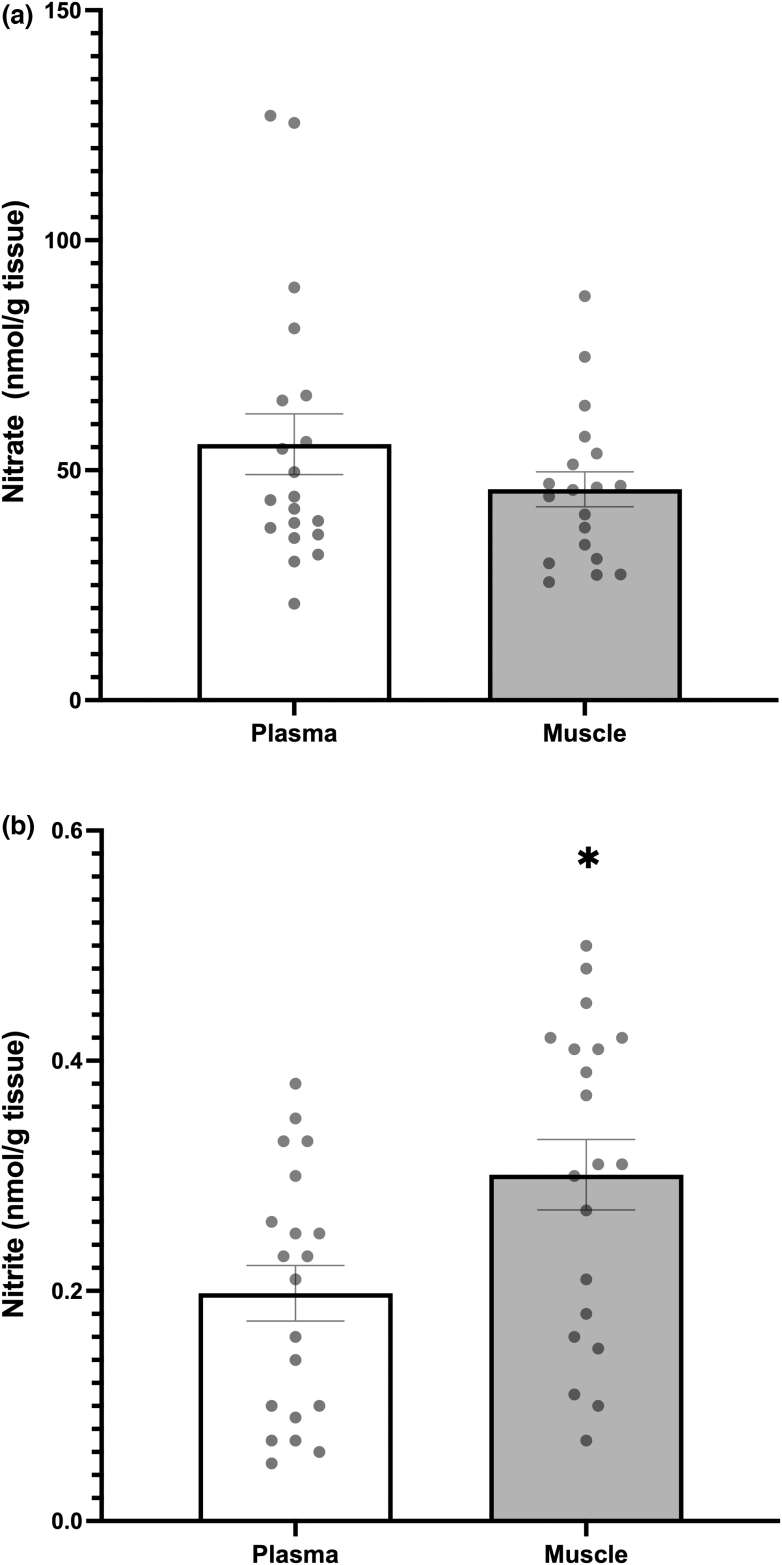
Baseline nitrate (panel a) and nitrite (panel b) in plasma (white Bar) and muscle (Gray bar). * difference between tissue within group (plasma vs. muscle, *p* < 0.05). Group means ± SEM.

The baseline (non‐supplemented) concentrations of nitrate and nitrite in both the plasma and skeletal muscle can be seen in Figure 2. There were no statistical differences in nitrate between plasma and muscle compartments (Figure 2a). However, for nitrite there was a small but statistically significant difference for nitrite between plasma (0.2 ± 0.11 nmol.g^−1^) and muscle (0.3 ± 0.14 nmol.g^−1^) (*p* = 0.02) (Figure 2b). Interestingly this finding was also present at the 12‐week samples in the participants with PAD who were assigned to PL treatment following randomization (0.16 ± 0.06 and 0.35 ± 0.18 nmol.g^−1^) for plasma and muscle nitrite, respectively (*p* = 0.01, supplemental data Figure S1).

### 3.2 The effect of dietary nitrate supplementation on muscle and plasma nitrate and nitrite concentrations at 12 weeks

**FIGURE 3:**
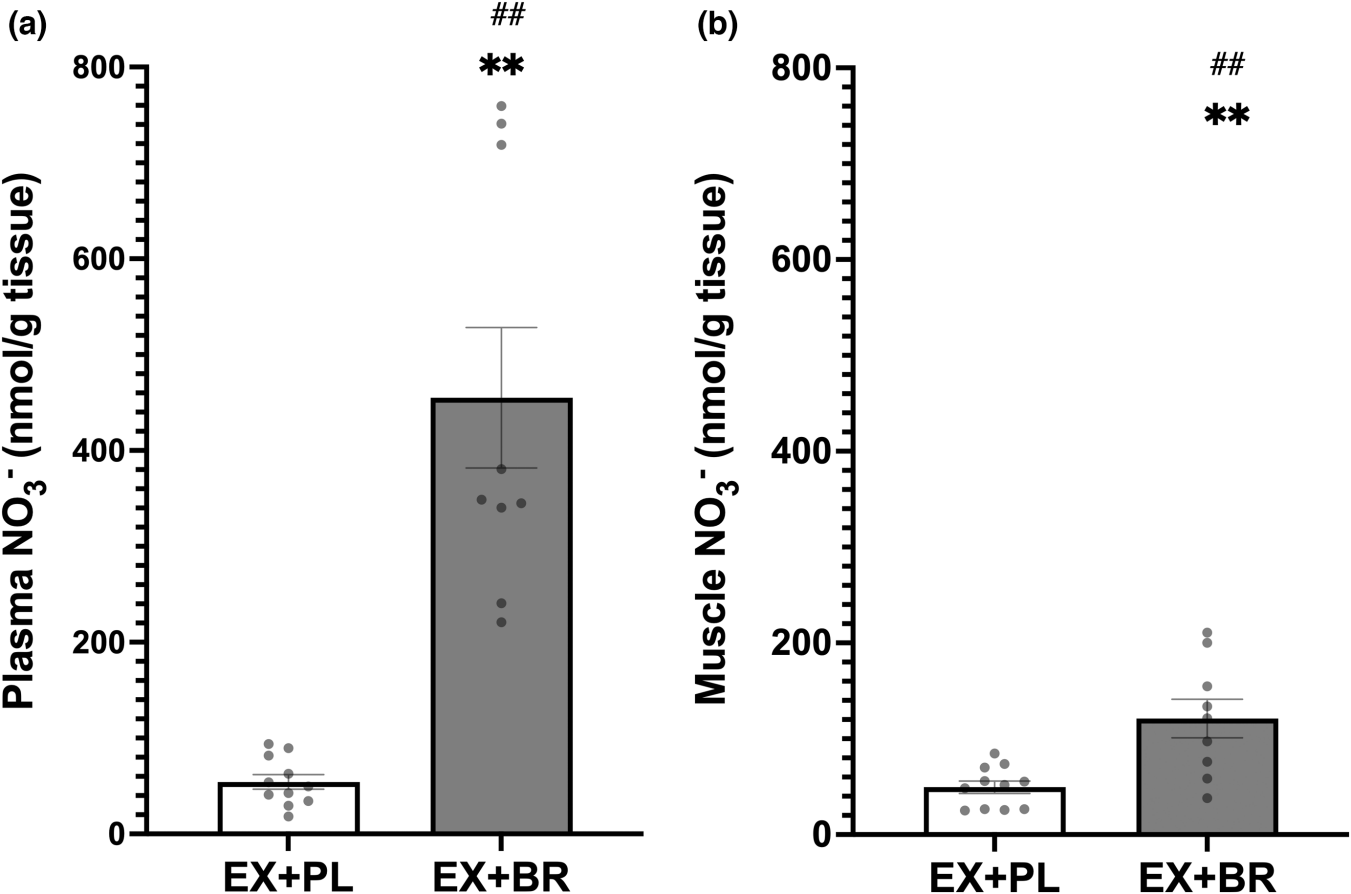
The concentration of nitrate in plasma (panel a) and muscle (panel b) following a 12‐week exercise training program in combination with either placebo (white bar) or beetroot juice (Gray bar). **difference within group (pre vs. post‐treatment, *p* < 0.01). *difference within group (pre vs. post treatment, *p* < 0.05). ^##^difference between groups (ex+PL vs. ex+BR) after supplementation (*p* < 0.01). Group means ± SEM.

Following BR supplementation, there were significant increases in both plasma and skeletal muscle nitrate from baseline (Figure 3). This also created a significant difference (or gradient) in nitrate concentrations in favor of plasma to muscle (455.04 ± 219.95 vs 121.14 ± 60.24 nmol.g^−1^, *p* < 0.01) respectively (see Figure S2 https://figshare.com/s/bea192ac8e6b24affef1).

**FIGURE 4:**
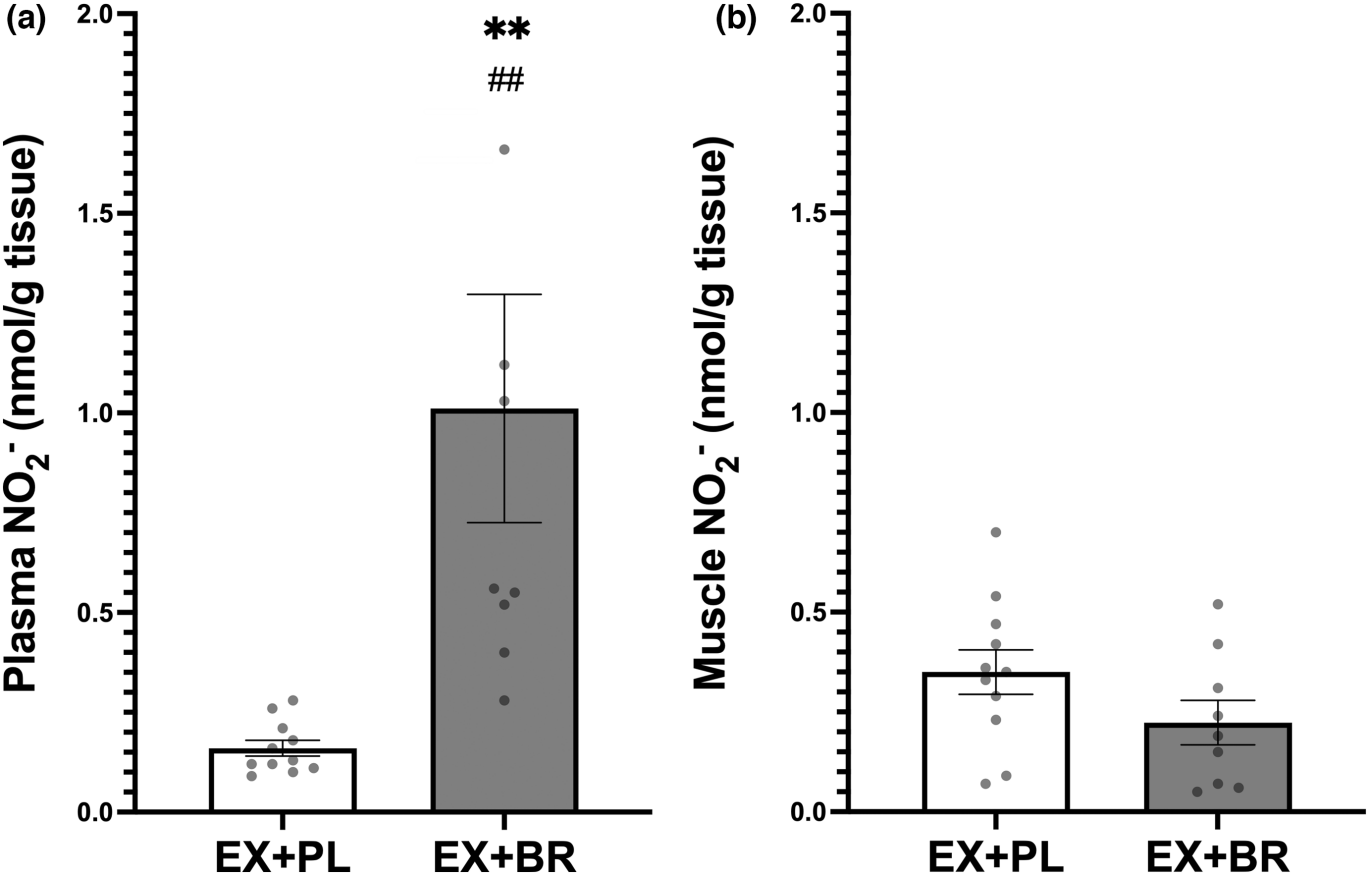
The concentration of nitrite in plasma (panel a) and muscle (panel B) following a 12‐week exercise training program in combination with either placebo (white bars) or beetroot juice (Black bars). **difference from pre‐supplement (PAD) (*p* < 0.01). ^##^difference between groups (ex+PL vs. ex+BR) after supplementation (PAD) (*p* < 0.01). Group means ± SEM.

Supplementation caused an increase in plasma but not muscle nitrite (Figure 4), which resulted in a significant difference (or gradient) between compartments in favor of plasma to muscle (1.01 ± 0.86 vs. 0.22 ± 0.17 nmol.g^−1^), (*p* < 0.05), respectively (Figure S2 https://figshare.com/s/bea192ac8e6b24affef1).

**FIGURE 5:**
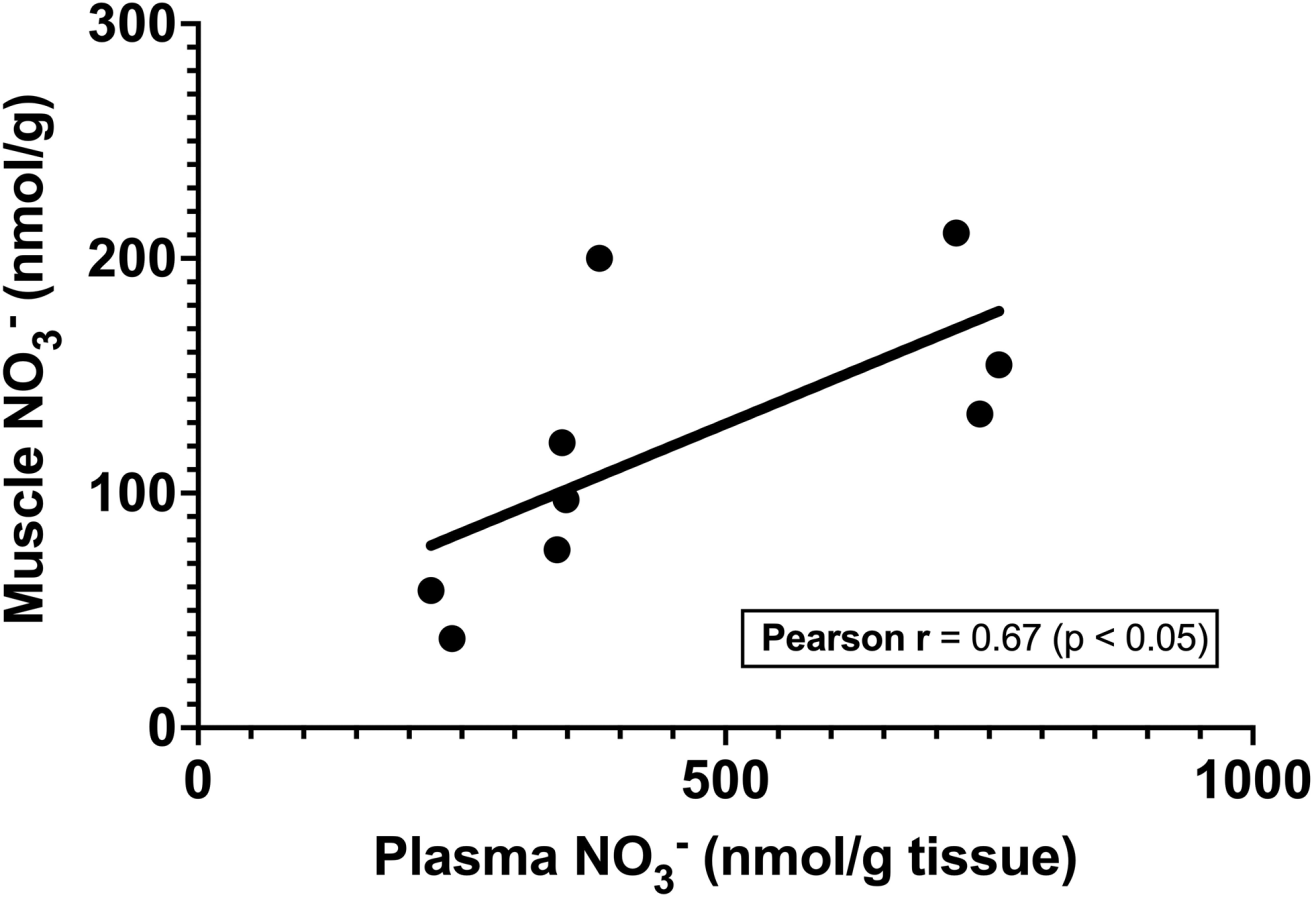
Relationship between plasma nitrate and muscle nitrate concentrations following BR supplementation.

There was a correlation between plasma and muscle nitrate concentrations following BR supplementation (Figure 5) (r = 0.67, *p* < 0.05). This was not the case for plasma and muscle nitrite concentrations which showed no relationship (not shown). In addition, there was no relationship between plasma nitrate and plasma nitrite (r = −0.14, *p* = 0.61), or muscle nitrate and muscle nitrite (r = 0.17, *p* = 0.50) values (data not shown).

### 3.3 Effect of dietary nitrate supplementation and exercise capacity

**FIGURE 6:**
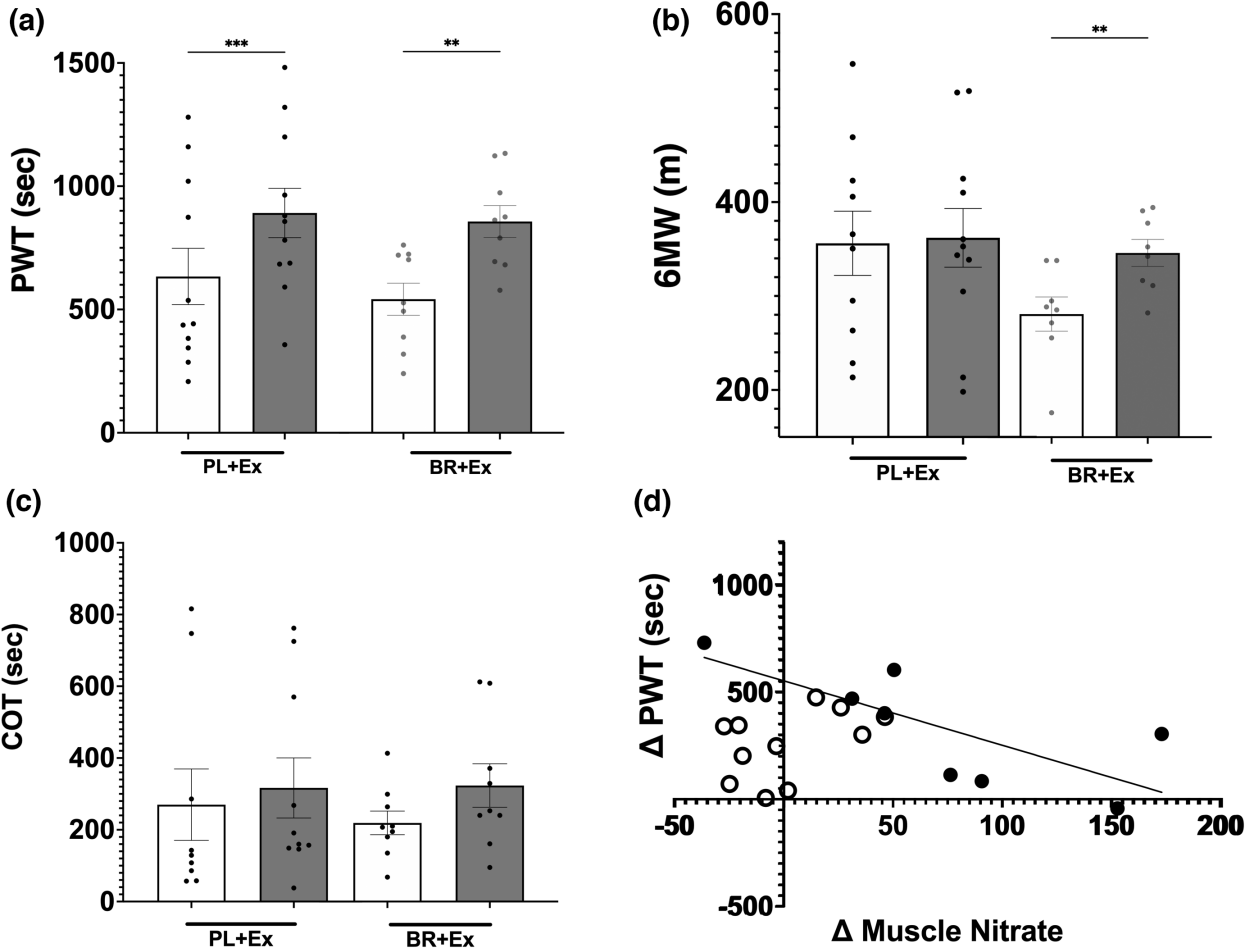
Exercise performance for treadmill peak walk time (PWT‐panel a), six minute walk distance (6 MW panel b); treadmill claudication onset time (COT‐panel c); relationship between skeletal muscle nitrate change and PWT change (panel d); after 12‐week exercise training program in combination with either placebo (EX+PL ‐ white circles) or beetroot juice (EX+BR ‐ Black circles). **difference from baseline (*p* < 0.01). Group means ± SEM.

There were no differences in exercise measures between BR and PL groups at baseline. Both treatment groups involved exercise training and both demonstrated increases in treadmill peak walk time (PWT) at the 12‐week timepoint (Figure 6a). Only participants assigned to EX+BR showed an increase in 6 MW distance (Figure 6b) along with a trend (*p* < 0.14) toward an increase (47.6%) in treadmill walking claudication onset time (COT) (Figure 6c). This value was significant in the larger parent study (Woessner et al., 2017).

The increase in skeletal muscle nitrate from baseline (non‐supplemented) to 12 weeks for the EX+BR group and acutely supplemented (Figure 3b) was negatively correlated with the change in treadmill PWT (r = −0.75, *p* < 0.05) (Figure 6d) but there were no correlations for any of the other nitrate or nitrite concentrations in plasma or muscle and exercise performance.

## 4 DISCUSSION

This is the first study to examine skeletal muscle nitrate and nitrite concentrations following dietary inorganic nitrate supplementation in participants with diagnosed PAD. We report, at baseline/pre supplementation no differences in nitrate between the plasma and the skeletal muscle tissues. The second major finding of this study is that supplementation with dietary inorganic nitrate in individuals with PAD, leads to significant increases in skeletal muscle nitrate but not skeletal muscle nitrite. Furthermore, there was a correlation between plasma and skeletal muscle nitrate concentration following BR supplementation suggesting a potential for communication between these compartments.

Both groups increased peak walking time following 12 weeks of exercise training but only those supplemented with BR increased 6 MW and tended to increase COT which may be more closely associated with the ischemic threshold and possibly increases in skeletal muscle capillary density and oxidative metabolism (Duscha et al., 2020). At 12 weeks following acute supplementation there was a negative correlation between increases in PWT and muscle nitrate concentration for the EX+BR group.

### 4.1 Baseline plasma and muscle nitrate and nitrite concentrations

At baseline testing (pre‐supplementation) there were no differences in nitrate concentrations between the plasma and skeletal muscle of participants with PAD. This is in agreement with findings in a previous report in healthy humans (Kadach et al., 2022) but contrasts with another report of concentrations 3‐fold higher in muscle than plasma (226 ± 213 vs. 54 ± 27 nmol.g^−1^, respectively, *p* < 0.01) (Wylie et al., 2019). In their study, the authors suggested that this gradient could allow the muscle tissue to act as a reservoir for N‐oxides.

Nitrite concentrations were statistically greater in the skeletal muscle than in plasma (Figure 2b) both at baseline for all subjects and in those randomized to EX+PL following supplementation (Figure S1 https://figshare.com/s/bea192ac8e6b24affef1). However, this gradient was small (0.10 nmol.g^−1^ and 0.18 nmol.g^−1^, respectively) in comparison to the gradients observed in young healthy individuals (~5.5 nmol.g^−1^) (Wylie et al., 2019). We can speculate that such differences could be a result of malfunctioning transporter proteins in PAD when compared with a healthy population.

It is possible that non‐supplemented muscle in PAD populations may not store or convert N‐oxides (nitrate and nitrite) eliminating a potential reservoir capacity that may be available in healthy young participants. This potentially reflects: (a) an inability to transport N‐oxides from the plasma to the muscle compartments; (b) a lack of NO production by skeletal muscle eNOS (NOS3) or nNOS (NOS1) (Piknova et al., 2015), due to their “uncoupling” (likely related to oxidation of NOS main cofactor, tetrahydrobiopterin or decreased availability of calcium ion (Cai & Harrison, 2000; Oceandy et al., 2007; Schuh et al., 2001)); (c) greater NO consumption by relatively lower oxygen tensions within the tissues (Elrod et al., 2008) as seen in tissue extracted from patients' incident leg who suffer from critical limb ischemia (Polhemus et al., 2015). Further studies examining of the expression/function of proteins involved in nitrate/nitrite/NO cycle are needed, to better understand these differences.

### 4.2 The effect of dietary nitrate supplementation on muscle and plasma nitrate and nitrite concentrations at 12 weeks

Following the 4.2 mmol nitrate dose (on the day of testing) in PAD, skeletal muscle nitrate increased by 77.27 nmol.g^−1^ (~3‐fold) above resting/PL levels. This skeletal muscle nitrate level is similar to those reported in participants with type 2 diabetes mellitus (T2D) and healthy young individuals following a single dose of 12.3 and 12.8 mmol potassium nitrate, respectively (Kadach et al., 2022; Nyakayiru et al., 2017). In comparison, a prior study in healthy young individuals following an acute 12.8 mmol dose of inorganic nitrate (via BR) increased skeletal muscle nitrate by 913 nmol.g^−1^ (a 5‐fold increase) (Wylie et al., 2019) (Figure 6). Further investigation may be able to better differentiate these effects.

The relatively lower values of skeletal muscle nitrate observed following supplementation, in individuals with PAD may be potentially due to a variety of factors including; lower muscle nNOS‐activity (Elrod et al., 2008); lower vascular eNOS‐activity; higher NO reduction by ROS; or reduced expression of membrane transport proteins, such as sialin (Dröge, 2003; Li et al., 2018; Yang et al., 2009). We did not explore the presence of sialin in the current study, but it is possible this transport protein maybe less abundant in clinical populations.

The current data also reported that the increased plasma nitrate was correlated with increased muscle nitrate following supplementation, suggesting the potential for movement between the blood and muscle at higher concentrations (Figure 5). Future studies should assess the presence of nitrate transport proteins in healthy and diseased populations to clarify these issues.

Despite significant increases in plasma nitrite following dietary nitrate supplementation, skeletal muscle nitrite levels did not change. This aligns with previous data from healthy humans that also showed no significant increase in muscle nitrite (Kadach et al., 2022; Wylie et al., 2019). The data in animal models are mixed, with both significant (Park, Thomas, Schechter, & Piknova, 2021) and non‐significant (Gilliard et al., 2018) increases in skeletal muscle nitrite following a 1 g/L sodium nitrate for 5–7 days, and following sustained release sodium nitrite (80 mg BID) in swine with metabolic syndrome and critical limb ischemia (Polhemus et al., 2015). Currently, our and other human data suggest a decreased transport of nitrite between the vascular compartment and the skeletal muscle, combined with possible limited nitrate reduction in muscle itself in participants with PAD.

Current data from studies in humans suggest that nitrate reduction to nitrite within skeletal muscle might be limited although oxidation from nitrite to nitrate is likely. Even though XOR has been confirmed to have the capacity to reduce nitrate to nitrite (Millar et al., 1998), a recent review highlights the required biochemical environment (i.e., oxygen tension, pH and XOR isoform) that determines XOR's nitrate‐ and nitrite‐reductase activity (Ortiz de Zevallos et al., 2022). Additionally, compared to rodents, humans have a relatively smaller expression of XOR within this tissue which could explain the differences between human and animal models results (Park, Thomas, Schechter, & Piknova, 2021). As such, humans might be able to store nitrate within skeletal muscle for subsequent reduction to nitrite through the entero‐salivary pathway and hepatic tissue rather than locally by XOR. These hypotheses need further investigation as current data is limited to draw conclusive statements on the role of skeletal muscle on the nitrate‐nitrite‐NO pathway as well as XOR's activity within this tissue.

### 4.3 Effect of dietary nitrate supplementation and exercise capacity

Inorganic nitrate supplementation has been shown to increase exercise performance in healthy participants and those with diagnosed cardiovascular disease, although the literature is relatively mixed and may depend on dosage, population, and activity. In participants with PAD, three studies (including the parent study here) show increases in ambulation (Bock et al., 2018; Kenjale et al., 2011; Woessner, VanBruggen, et al., 2018). The current data show increases in 6 MW distance and COT (trend) for the EX+BR group and extend our previous findings (Woessner, VanBruggen, et al., 2018) by suggesting chronic nitrate supplementation and the corresponding increases in skeletal muscle nitrate could potentially promote long term changes in the ischemic threshold which is likely mediated via increases in skeletal muscle capillary density and oxidative metabolism (Duscha et al., 2020). This could be associated to the increased exercise capacity after dietary nitrate supplementation in each training session allowing patients to have larger localized training stimulus (Muggeridge et al., 2017). It has also been suggested that combining dietary inorganic nitrate and training might promote improved aerobic metabolism by shifting glycolytic fibers to a more aerobic phenotype which have been associated with delayed accumulation of exercise metabolites within skeletal muscle (Thompson et al., 2017). However, no between group difference in PWT (both groups improved) and the negative relationship PWT change and skeletal muscle nitrate concentration change for the EX+BR group suggests no role for increased muscle nitrate to acute exercise performance in PAD. This extends the finding in healthy humans who found no increase in acute exercise performance despite increased skeletal muscle nitrate concentrations (Wylie et al., 2019), however both of these studies may have been underpowered for this outcome variable. Additionally, this brings new research questions about supplementation regimes and dosing in clinical populations as it has been recently shown that higher NO bioavailability does not necessarily result in better skeletal muscle performance (Gallardo et al., 2021).

### 4.4 Study limitations

Our study has several limitations of note. First, our sample size is relatively small, as it is comprised of a sub‐group from a larger clinical trial. There is an established inter‐ and intra‐ individual variation in the nitrate and nitrite levels and the small sample size limits our ability to generalize the findings to larger cohorts. Also, the present PAD study sampled skeletal muscle from gastrocnemius and the cited healthy comparator studies used vastus lateralis (Kadach et al., 2022; Wylie et al., 2019). While the results for nitrate/nitrite and correlations were similar across both muscles, we cannot rule out the possibility that the muscle type and fiber composition could have an impact on the findings (Long et al., 2020; Park, Thomas, Schechter, & Piknova, 2021). Previous work in rodent muscle has shown significantly different basal levels of nitrate and nitrite between predominantly fast and slow twitch fiber types, although all had increased levels of nitrate/nitrite with supplementation (Park, Thomas, Schechter, & Piknova, 2021). However, human muscles have less distinct differentiations of fiber type (more heterogenous types within a muscle) than rats potentially reducing the influence of the particular muscle selected.

Finally, subjects were on treatment (BR or PL) on the days of muscle biopsies and exercise testing and had undergone blood sampling on repeated days to test for consistency of plasma concentrations. Biopsies were performed on a separate day to the exercise testing. We did not directly measure the variations between these blood and muscle compartments over a prolonged period following supplementation. Interestingly, recent data in healthy young individuals suggests nitrate in the muscle, plasma and saliva may all follow a similar pattern with levels rising rapidly up to 2.5 h post administration before returning to baseline by 9 h (Kadach et al., 2022). Our correlations suggest that nitrate may be transported between compartments, but future studies should explore this mechanism more directly to confirm these findings and explore possible differences between healthy people and patients with cardiovascular diseases.

## 5 CONCLUSION

**FIGURE 7:**
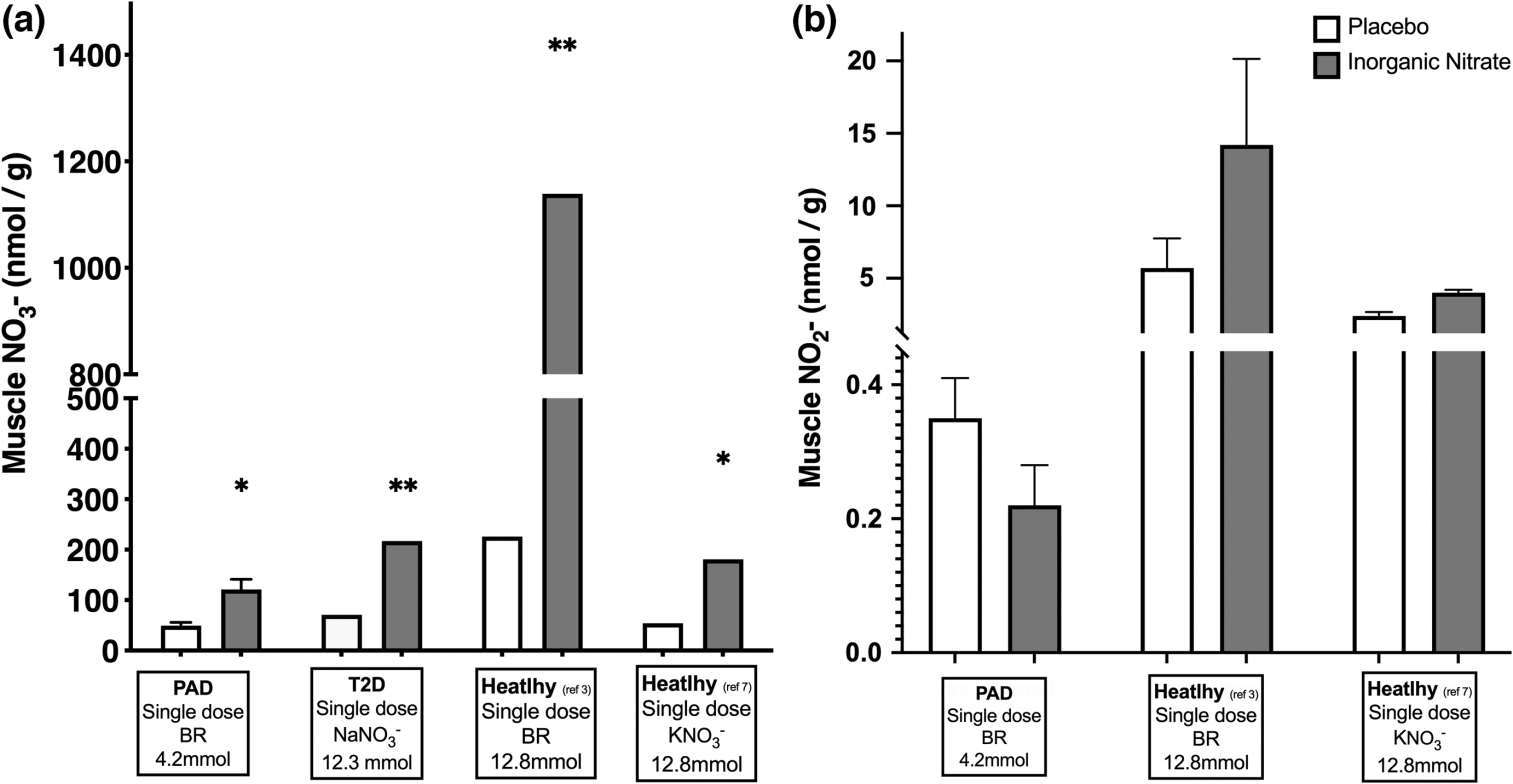
Nitrate (panel a) and nitrite (panel b) storage and metabolism in different populations. Data for T2D has been adapted from Nyakayiru et al (2017). Data for healthy individuals has been adapted from Wylie et al (2019) and Kadach et al (2022). Nyakayiru et al (2017) did not report skeletal muscle nitrite concentrations at baseline or after supplementation. *different from placebo (*p* < 0.05). **different from placebo (*p* < 0.01).

**FIGURE 8:**
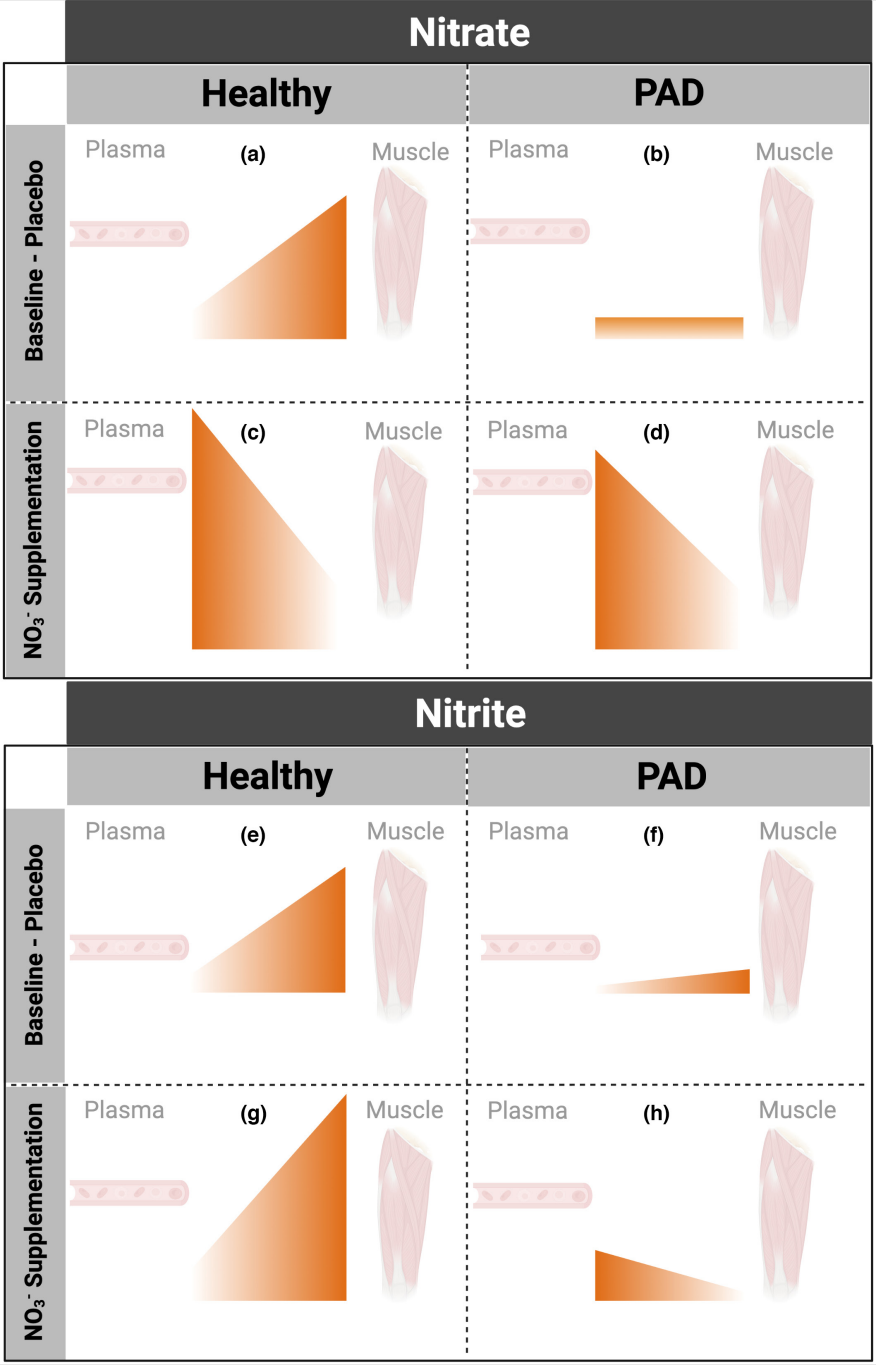
Schematic representation showing a comparison of plasma and skeletal muscle nitrate (panels a–d) and nitrite (panels e–h) concentrations in healthy young subjects (previously reported) and individuals with peripheral arterial disease (PAD) at baseline/placebo (panels a, b, e, f) and following oral inorganic nitrate supplementation (panels c, d, g, h).

The present study is the first to show that, at baseline, no substantial gradient of nitrate (or nitrite) between plasma and muscle exists in participants with PAD, which is in contrast with observations in healthy individuals (Figure 7). This suggests limited skeletal muscle NOS‐derived NO production/oxidation or nitrate storage in this condition. Oral inorganic nitrate supplementation leads to increases in skeletal muscle nitrate suggesting potential “nitrate concentration exchange” between plasma and skeletal muscle. This idea is supported by the positive correlation between these two compartments after supplementation. In contrast, skeletal muscle nitrite does not increase after supplementation suggesting that nitrite may be less able to readily move between compartments and that nitrate is unlikely to be able to be reduced to nitrite within the muscle. Accumulation of N‐oxides within skeletal muscle did not appear to play a role in improving exercise performance in PAD. Based on our findings and previous reports in healthy individuals (Figure 7) we have drawn a cartoon (Figure 8) to summarize the current literature in this area.

Future studies should focus on further elucidating the explicit role the muscle reservoir could play in increasing NO bioavailability in health and disease and further optimize our dosing strategies for health and functional benefits.

## AUTHOR CONTRIBUTIONS

The experiments on human participants, including muscle biopsy procedures, were performed at Duke University Medical Center, USA. The tissue analyses were completed at the US National Institutes of Health. Barbora Piknova, Joaquin Ortiz de Zevallos, Alan N. Schechter, and Alan N. Schechter. contributed to the conception and design of the work; Barbora Piknova, Mary N. Woessner, Joaquin Ortiz de Zevallos, William E. Kraus, Mitch D. VanBruggen, Alan N. Schechter, and Alan N. Schechter contributed to the acquisition, analysis, or interpretation of data for the work; Barbora Piknova, Mary N. Woessner, Joaquin Ortiz de Zevallos, William E. Kraus, Mitch D. VanBruggen, Alan N. Schechter, and Alan N. Schechter all contributed to drafting the work or revising it critically for important intellectual content. All authors approved the final version of the manuscript and agree to be accountable for all aspects of the work in ensuring that questions related to the accuracy or integrity of any part of the work are appropriately investigated and resolved. All persons designated as authors qualify for authorship, and all those who qualify for authorship are listed.

## FUNDING INFORMATION

The PAD work was supported by grants to J.D. Allen from the National Heart, Lung, and Blood Institute R21HL111972 and R21HL113717.

## CONFLICT OF INTEREST

Alan N. Schechter is listed as a co‐inventor on several patents issued to the National Institutes of Health for the use of nitrite salts for the treatment of cardiovascular diseases. He receives royalties based on NIH licensing of these patents for clinical development but no other compensation. The other authors declare that they have no conflicts of interest.

## ETHICS STATEMENT

Institutional Ethics Committee Approval number: Duke University Institutional Review Board protocol# Pro00031918 and Pro00039608.
